# An alternative conformation of ERβ bound to estradiol reveals H12 in a stable antagonist position

**DOI:** 10.1038/s41598-017-03774-x

**Published:** 2017-06-14

**Authors:** Paulo C. T. Souza, Larissa C. Textor, Denise C. Melo, Alessandro S. Nascimento, Munir S. Skaf, Igor Polikarpov

**Affiliations:** 1 Institute of Chemistry, University of Campinas - UNICAMP, P. O. Box, 6154 Campinas, SP Brazil; 2 Groningen Biomolecular Sciences and Biotechnology Institute, University of Groningen, Nijenborgh 7, 9747 AG Groningen, The Netherlands; 3 Zernike Institute for Advanced Materials, University of Groningen, Nijenborgh 7, 9747 AG Groningen, The Netherlands; 4 São Carlos Institute of Physics, University of São Paulo - USP, P.O. Box 396, São Carlos, SP Brazil

## Abstract

The natural ligand 17β-estradiol (E2) is so far believed to induce a unique agonist-bound active conformation in the ligand binding domain (LBD) of the estrogen receptors (ERs). Both subtypes, ERα and ERβ, are transcriptionally activated in the presence of E2 with ERβ being somewhat less active than ERα under similar conditions. The molecular bases for this intriguing behavior are mainly attributed to subtype differences in the amino-terminal domain of these receptors. However, structural details that confer differences in the molecular response of ER LBDs to E2 still remain elusive. In this study, we present a new crystallographic structure of the ERβ LBD bound to E2 in which H12 assumes an alternative conformation that resembles antagonist ERs structures. Structural observations and molecular dynamics simulations jointly provide evidence that alternative ERβ H12 position could correspond to a stable conformation of the receptor under physiological pH conditions. Our findings shed light on the unexpected role of LBD in the lower functional response of ERβ subtype.

## Introduction

Estrogen receptors (ERs) are transcription factors involved in several biological processes, such as the growth, development and cell differentiation, including important regulatory functions within the reproductive and central nervous systems, mammary glands, and bone tissues, among others. Furthermore, ERs are important targets for the treatment of osteoporosis, breast and uterine cancers. Their cognate hormone, 17β-estradiol (E2) is widely used in hormone replacement therapies and oral contraceptives^1–4^. Similar to other members of the nuclear receptor (NR) superfamily, ERs are formed by a modular structure composed of a variable amino-terminal domain (NTD) that encloses a ligand-independent activation function (AF-1), a central conserved DNA-binding domain (DBD), a flexible linker or hinge region, a ligand-binding domain (LBD) encompassing the ligand-dependent activation function (AF-2), and an extended carboxy-terminal F domain, which has been shown to modulate the gene transcription activation^4^.

Allosteric modulation through LBD conformational dynamics is a crucial process for the ligand-dependent transcriptional regulation since this domain is responsible for the primary cascade of events, including hormone recognition, receptor dimerization and structural rearrangement of AF-2 that modulate corepressors and coactivators binding. The ligand-dependent differential recognition of these coregulators by the AF-2 hydrophobic groove is closely related to the conformation of the helix 12 (H12)^5, 6^. A number of studies of apo and ligand-bound NR LBDs have shown that this domain in general and H12 in particular, are usually more mobile and unstructured in the absence of ligand^7–12^. Upon ligand binding, the dynamic globular structure is rearranged and stabilized, shifting the equilibrium of H12 conformations towards a specific low energy state. While agonist binding induces the packing of H12 against H3 and H5 creating the hydrophobic binding surface for coactivator binding and leading to concomitant corepressor release, antagonists prevent the repositioning of H12 into the active conformation and, consequently, coactivator recruitment^13, 14^. The currently accepted hypothesis supports a graded agonism model where activity levels reflect the population of conformers having H12 in the active conformation^8, 15^. This view is exemplified by crystallographic structures of ER LBD associated with the partial agonist WAY-169916, where both active and inactive conformations could be induced by mutations^15^.

There are two subtypes of ER, ERα and ERβ, which are encoded by different genes in separate chromosomes^16^. The primary structure and the modular organization of the subtypes are similar, with ERα (595 residues) being larger than the ERβ (530 residues). ERs DBDs have very high homology (96%), while ERs LBDs are only about 59% homologous^17^, despite the high similarity between the tertiary structures, particularly in the coactivador/corepressor pocket of the AF-2 region (100% conserved) and the buried ligand-binding pocket (LBP), that differ by only two amino acid residues: M336 (ERβ) is replaced by L384 (ERα) in H6 helix and I373 (ERβ) is substituted by M421 (ERα) in H8 helix^18^. These substitutions in the LBP do not result in significant differences in E2 affinity^19^, but can be important for the development of selective ligands^3^. The main differences between subtypes are in the NTDs, which share only 30% of sequence identity, with ERβ AF-1 being shorter than that of ERα^17^.

It is well established that both subtypes are transcriptionally activated in the presence of E2, with ERβ being somewhat less active than ERα under similar conditions^20, 21^. Thus, in tissues where both receptors are expressed, ERβ is found to inhibit ERα-mediated gene transcription^22^. In general, these functional activity profiles are only attributed to the “weaker” ERβ AF-1 region. However, some studies clearly show that AF-1 differences alone are not sufficient to explain these behaviors. For example, a chimera containing the ERα NTC domain and the remaining structure of ERβ (DBD, hinge, LBD and F-Domain) does not fully recover the E2-dependent activity of the wild-type ERα, thus indicating that the differences in ERβ LBD could also be important^21^. In fact, a similar situation is observed for another steroid receptor, the mineralocorticoid receptor that binds both mineralocorticoids and glucocorticoids with a similar affinity. It has been shown that both pre- and post-receptor mechanisms play important roles in ligand selectivity, but relevant roles are also played in the receptor level by the ligand binding domain^23^.

The molecular basis for this intriguing behavior, known as the “Yin-Yang” relationship between ERβ and ERα, remains elusive, especially in regard to the role of the LBD. In this study, we present a new crystal structure of human ERβ LBD bound to E2, where the C-terminal H12 is packed towards the receptor in an alternative conformation that closely resembles that of ER (and other NRs) LBDs bound to antagonist ligands. Our structural analysis and molecular dynamics (MD) simulations of ER subtypes and mutants provide evidence that H12 of the ERβ LBD is not as stable as ERα LBD H12 in its active canonical conformation. Furthermore, extensive (3.5 microseconds) MD simulations were carried out to map the free-energy landscape across the conformational space spanning both the canonical and the newly determined ERβ LBD-E2 liganded structures. Our results suggest that the protonation state of the His 498 can determine which of the two structures is stabilized in solution. The alternative ERβ H12 conformation should correspond to a stable LBD conformation under normal physiological pH conditions. These findings help to explain the molecular reasons for the lower activity of ERβ subtype, elucidate the role of LBD in the Yin-Yang relationship between ER subtypes, and may contribute to the development of tissue-selective modulators of ER activation for pharmaceutical applications.

## Results and Discussion

### An alternative structure of ERβ LBD bound to E2 reveals H12 in an antagonist-like conformation

**Figure 1:**
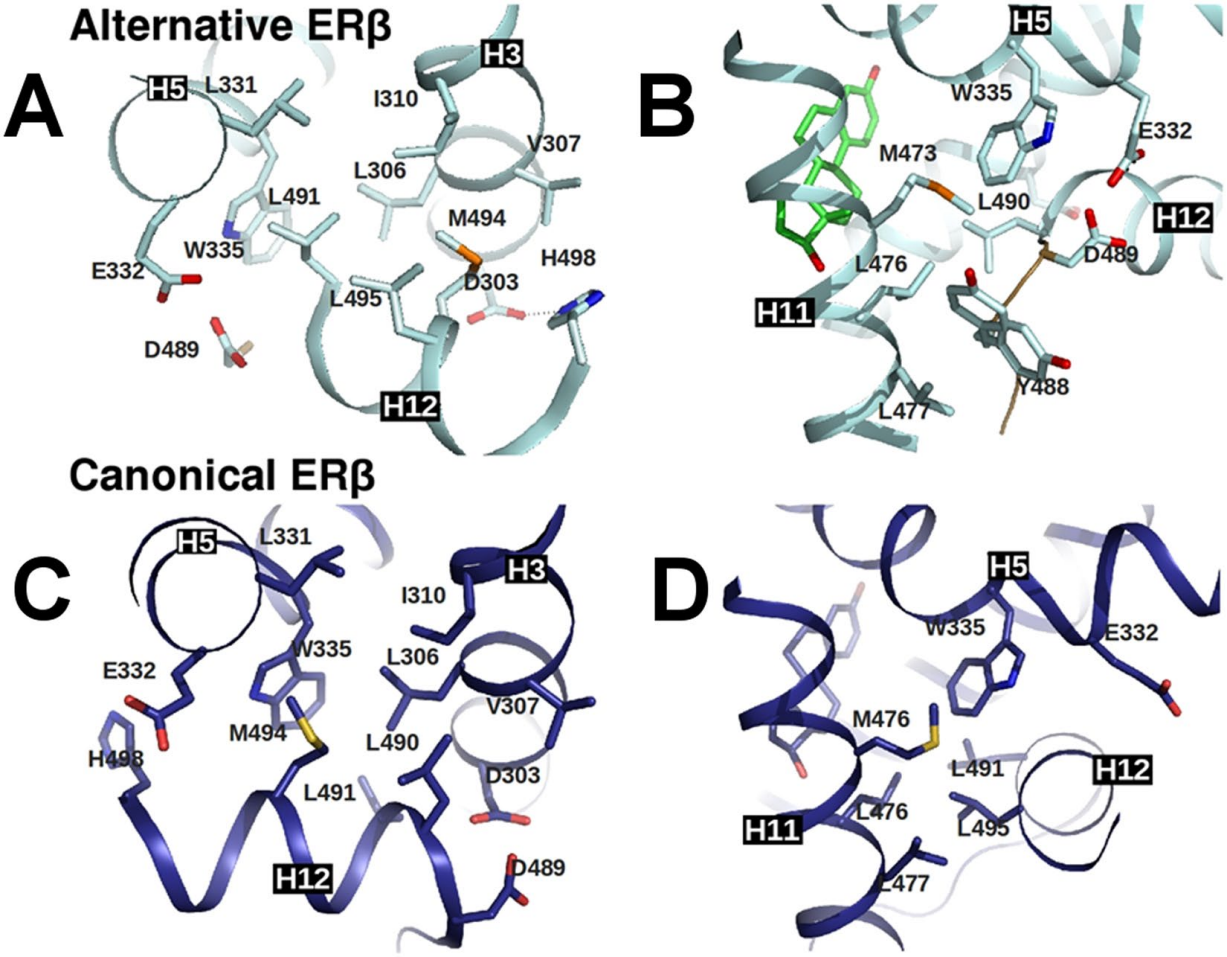
H12 contacts of alternative and canonical ERβ LBD-E2 structures: Schematic representation of the H12 packing against the LBD of alternative ERβ-E2 (with LBD in cyan and E2 in green) in comparison to the canonical ERβ-E2 (blue, PDB code 3OLL) showing the residues involved in the interactions of H12 with H3, H5 and H11. L491 of alternative structure (H12 helix, in center of Fig. 1A) packs against I310 (H3), L331 (H5) and W335, which is similar to the interactions of M494 of canonical ERβ-E2 (H12, in Fig. 1C). The carboxy-terminal part of alternative H12 (in Fig. 1A) moves towards H3 and make contacts at this helix with L306 and V307 via M494. These contacts are comparable to L490 in canonical ERβ LBD-E2 (Fig. 1C). The side chain of L490 in the alternative H12 (as shown in Fig. 1B) occupies an intermediate position of L491 and L495 in canonical ERβ LBD-E2 structure (Fig. 1D) and similarly packs against W335 (H5) and L476 (H11).

The ERβ LBD-E2 crystallographic structure that we determined displays the classical three-layered α-helical sandwich fold, resembling the known ERs LBD structures^13, 15, 24–26^, except for AF-2 region. The C-terminal H12 lays over H3, H5 and H11 covering part of the LBP via its hydrophobic side, as shown in Fig. 1. However, H12 is oriented in opposite direction to the one usually seen in all active agonist-bound structures of ER LBDs. The residues involved in the hydrophobic interactions between H12 and the remaining LBD are basically the same as in the already published structures of human ERβ LBD bound to E2 (called here canonical ERβ LBD-E2 structure)^25^, though the pairwise inter-contacting patterns are not maintained due to the direction adopted by H12 in the current structure.

**Figure 2:**
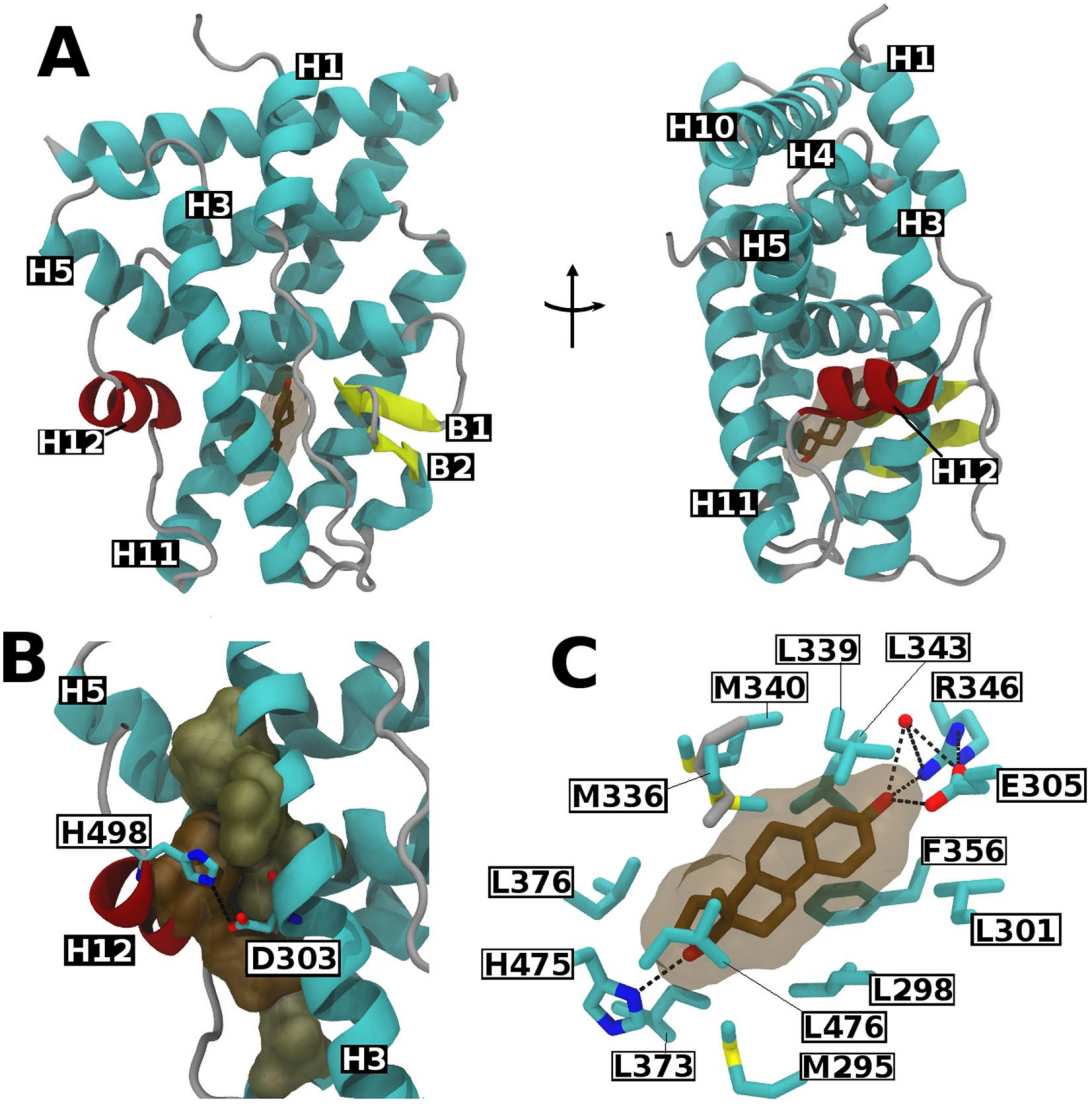
Alternative Structure of the ERβ LBD in complex with E2: (**A**) Ribbon representations of the ERβ LBD-E2 crystallographic structure indicating the locations of the α-helices (H, cyan), β-sheets (**B**) yellow), coil regions (gray) and E2 (brown surface) in its ligand-binding avity. Helix 12 in its alternative position is highlighted in red. (**B**) Interactions between H12 and the remaining LBD. Hydrophobic contacts are represented as brown surfaces and the hydrophilic interaction D303-H498 as a black dashed line. (**C**) Schematic representation showing E2 (brown) and its interactions with the residues of ERβ LBD (cyan). Hydrophilic interactions are represented by black dashed lines. The only observed difference of E2 binding mode of our structure and the already published structures of ERβ LBD-E2 (PDB codes 3OLL and 3OLS) is the position of the M336 side chain (highlighted in gray).

In addition to the hydrophobic contacts (Fig. 1), the main molecular interaction that seems to be responsible for directing the alternative positioning of H12 (Fig. 2A) is the polar interaction between D303 in H3 and H498 in H12 (Fig. 2B). Thus, despite the different orientation, the alternative H12 conformation seals the highly hydrophobic LBP in a similar manner to the canonical conformation. As a consequence, similar interactions would be expected in the binding of E2 for both structures.

In fact, E2 is completely buried in the LBD and its position, as well as the interactions involved in its recognition, are absolutely consistent with the previous data from the canonical ERβ LBD-E2 structure^25^. Figure 2C shows all the residues that contact E2 in the alternative structure conformation. The residues M295, L298, and L301 in helix H3; M336, L339, M340, and L343 in helix H6; F356 from the β-sheet B1; L373 and L376 from helix H8; and also L476 in H11 make hydrophobic interactions with the ligand. Furthermore, similar to other ER LBD-E2 structures, there are two groups of hydrophilic interactions with the ligand: i) the phenolic hydroxyl of the E2 A-ring makes direct hydrogen bonds to the carboxylate of E305 (H3), the guanidinium group of R346 (H6), and/or a water molecule; and ii) the 17-β hydroxyl of E2 D-ring makes a hydrogen bond with H475 from H11. The only observed difference between the LBPs in the canonical and alternative conformations is the position of the M336 side chain, as highlighted in Fig. 2C. This difference does not seem to implicate in any loss of hydrophobic contacts of M336 with E2.

**Figure 3:**
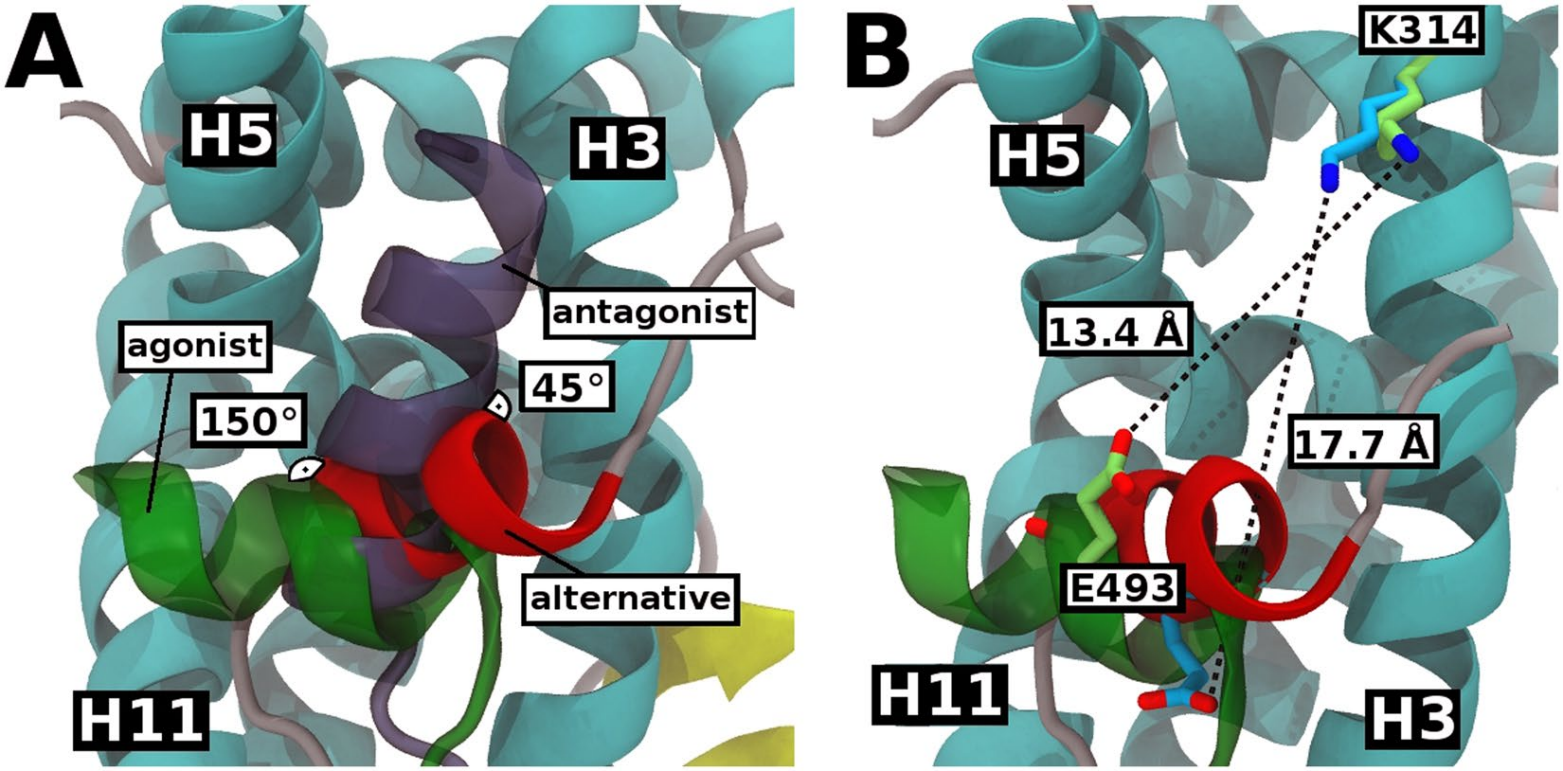
Comparison of the alternative, canonical agonist and antagonist bound structures: (**A**) Angle displacement between H12 position of alternative ERβ LBD-E2 (red), canonical agonist ERβ LBD-E2 (green, PDB code 3OLS) and antagonist ERβ LBD-genistein (purple, PDB code 1QKM) structures. Alternative structure appears to be in a similar position to the antagonist structure. (**B**) Distance between the charge-clamp residues (K314 and E493) of alternative ERβ LBD-E2 (H12 in red, residues in cyan) and canonical agonist ERβ LBD-E2 (H12 and residues in green, PDB code 3OLS). The charge clamp of canonical conformation is an optimal distance, since the structure was associated with a coactivator peptide.

The H12 alternative conformation observed in the present structure raises the question about what would be its role in the ERβ activity if this new conformation remains stable in solution. The similarities between the alternative conformation and the active and inactive canonical conformations of ER LBDs provide an answer. Figure 3A shows a comparison between the H12 position of the current structure and the published structures of human ERβ LBD bound to E2^25^ and to genistein^18^. Surprisingly, its position seems to be more similar to the antagonist structures such as ERβ LBD-genistein complex determined in the absence of coactivator. The displacement angle was approximately 150° and 45° in comparison to the canonical agonist and antagonist H12 positions, respectively. H12 RMSD reflects differences in its orientations, with the alternative position displaying a deviation of 8.8 Å with respect to the canonical H12 backbone (residues 489 to 497) while deviating from the antagonist conformation by only 5.7 Å (canonical and antagonist H12 conformers have RMSD of 9.6 Å between themselves). Despite the fact that the coactivator-interaction groove partially overlaps with H12 in the alternative conformation (Supplemental Figure S1), careful analysis of the structure shows that the distance between the “charge clamp” residues (K314 and E493) is larger than in the structures associated with coactivators (Fig. 3B). The charge-clamp residues interact with both termini of the coactivators α-helical LXXLL motif, being important for its proper positioning and recognition by NR LBDs^27^. In the present structure, the 150° rotation of H12 relative to the canonical active conformation takes the E493 residue completely out of its optimal position for the recognition of the coactivators LXXLL motif. Thus, the alternative ERβ LBD-E2 conformation should block association with coactivators, being more similar to an antagonist-like conformation.

The main crystallization condition difference, which led to the alternative and canonical ERβ LBD-E2 structures (Fig. 3B) is that the alternative structure was obtained in the absence of the coactivator LXXLL peptide. In contrast, the previously published ERα LBD-E2 structures exhibit H12 in the same canonical position, both with and without coactivators peptides^28^. These subtype structural differences suggest that in the absence of coactivators, the H12 canonical position of ERβ LBD may not be as stable as in the ERα subtype. Interestingly, ERβ LBD structures bound to the partial agonist genistein show a similar pattern to ERβ LBD-E2: a canonical H12 conformation in the presence of coactivator peptide and an antagonist-like conformation in its absence^18, 29^. Thus, in terms of the structural arrangement of H12, the similarity with genistein might indicate that E2 may also play the role of a partial agonist of ERβ. The question then arises as to under what circumstances would the agonist (canonical) or antagonist character of E2 on ERβ prevail.

### The lack of H12 stability in a canonical conformation of ERβ LBD: a new look at the published crystallographic structures using MD simulations

The stabilization of a particular H12 conformation depends on the interactions between its residues, bound ligands, coactivators and the remaining LBD. Curiously, E2 does not make direct contacts with H12 in either ER subtypes (see Fig. 1). Its effect on stabilizing the AF-2 region is indirect, through stabilization of H3, H11 and H5/6 around the LBP^30^. Therefore, in the absence of coactivators, the interactions with the remaining LBD are primarily responsible for stabilizing the H12 position. The canonical structures of ERs LBD bound to agonist ligands are very similar, despite their sequences being just 59% identical^17^. Obviously, the H12 backbone positions in both subtypes are nearly identical. However, the contacts of H12 with LBD are not as similar.

**Figure 4:**
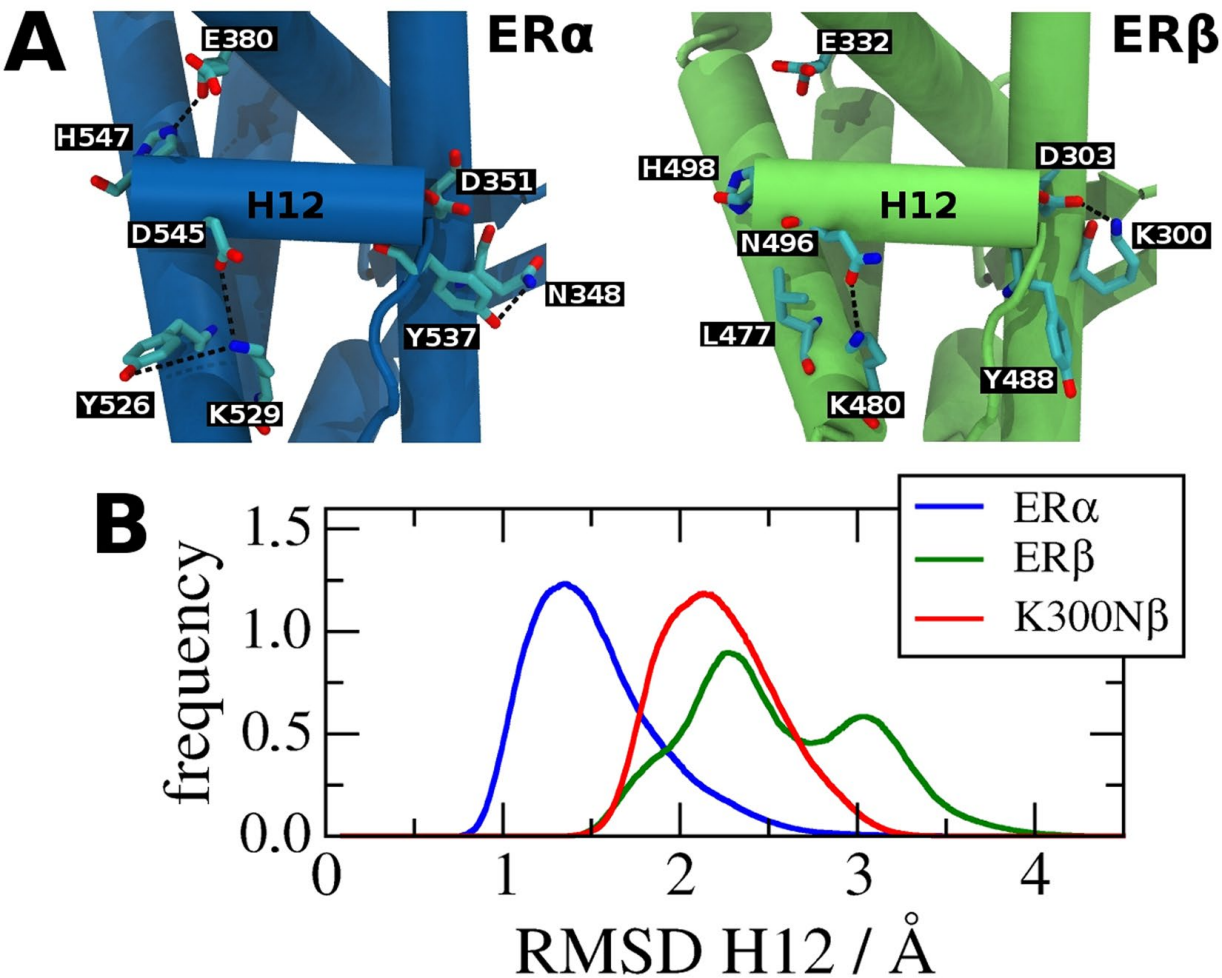
Stability of H12 canonical position in ER subtypes: (**A**) Comparison of main interactions between H12 and the remaining canonical LBD structures of the ERα LBD-E2 (blue, PDB code 1QKU) and ERβ LBD-E2 (green, PDB code 3OLS). ERα LBD-E2 has three hydrophilic interactions stabilizing H12 in its canonical position while ERβ LBD-E2 has only one interaction. The position of histidine H498 was not obtained in 3OLS structure. Its position was added based on the structure of the phosphorylated ERβ LBD-E2 (PDB code 3OLL). (**B**) RMSD distribution of H12 computed with respect to MD simulation average structures of wild-type ERα LBD-E2 (blue), wild-type ERβ LBD-E2 (green) and K300N mutant of ERβ LBD-E2 (K300Nβ). H12 of wild-type ERα shows a lower mobility than wild-type ERβ. K300Nβ corresponds to the transformation β → α of one of the residues that stabilizes H12. As expected, this mutation reduces the mobility of this helix.

Figure 4A shows the main H12 hydrophilic interactions for both receptor subtypes. H12 in ERα LBD-E2 has three important interactions with helices H3, H5, and H11. The interactions between H11 and H12 involve the Y526, K529, and D545 residues. Y526 from H11 interacts with K529, which in turn also interacts with H12 through D545. One of these interactions, Y526-K529, locks the lysine side chain in a favorable position for the K529-D545 interaction, stabilizing the H12. In addition, the carboxy-terminus of H12 makes a second interaction involving E380 (H5) and H547 (H12). The third interaction takes place at the other end of H12, between residues N348 (H3) and Y537 (H12). The importance of residue Y537 for the ERα transcriptional activity had already been proven by mutation and phosphorylation assays at this particular site^25, 31–33^.

The canonical ERβ LBD-E2 structure clearly shows that H12 hydrophilic contacts with the remaining LBD are very different to those found in the α subtype, despite of the same agonist position. First, there is only one hydrophilic interaction with H12 in ERβ. This interaction occurs between K480 (H11) and N498 (H12), and is equivalent to the interaction K529 - D545 in ERα subtype. Furthermore, K480 should be fully available to the solvent in ERβ because the leucine L477 (ERβ) that substitutes Y526 (ERα) makes no direct interaction with K480. These alterations in ERβ render the interaction K480-N496 weaker than the analogous interaction in ERα. The other two observed interactions in ERα LBD-E2 have no equivalent in ERβ LBD-E2. The H498 position is frequently undefined in the crystal structures of the canonical ERβ LBD-E2, but was determined in a crystal structure of the phosphorylated ERβ LBD^25^ and in a crystal structure of ERβ LBD in complex with the ligand KB095285 and a peptide from the coactivator CIA12 (PDB id: 4ZI1). In these two structures, the position of H498 is directed toward the solvent, with no H498-E332 interaction (equivalent to E380-H547 in ERα), shown in Fig. 4A.

Similarly, the Y488 residue (analogous to ERα Y537) establishes no contact with H3 in ERβ. In this case, the substitution of an asparagine in ERα (N348) by a lysine (K300) of the ERβ subtype seems to be responsible for the loss of this interaction. Unlike the N348 residue in ERα, K300 makes an intrahelical interaction with D303 (H3) instead of the tyrosine on H12.

The structural differences between the subtypes strongly suggest that H12 should be less stable in ERβ than in ERα. Furthermore, specific modifications (point mutations or phosphorylation) in ERβ could restore part of H12 stability. To test these hypotheses, we compared the H12 mobility of the wild-type (wt) ERα, wt ERβ and K300N ERβ mutant obtained from MD simulations of their E2-liganded LBDs in the absence of coactivator peptides. As an indicator of mobility, we use the distribution of the H12 root mean square deviation (RMSD) relative to its average position. The results are shown in Fig. 4B. ERα H12 is the least mobile of the three cases, with the lowest values of RMSD and a narrower distribution. ERβ H12, in contrast, is the most mobile, consistent with the fact it makes fewer hydrophilic interactions with the remainder of the LBD. The mutant K300Nβ corresponds to one of the substitutions that we advocate would restore at least in part the H12-LBD interactions observed in ERα that are absent in ERβ, namely Y488 and N300. As anticipated in the light of the discussion above, the K300N mutation does reduce the H12 mobility compared to wt ERβ, as shown in Fig. 4B. This effect arises from increased interactions that take place between N300 and Y488 (ion-dipole) in the mutant and the concomitant loss of the intrahelical salt bridge with D303 due to asparagine’s shorter side chain compared to lysine’s. Interestingly, Tharun and colleagues recently identified the importance of K300 in the case of the phosphorylated ERβ^33^. They observed that phosphorylation of Y488 enhances the ERβ affinity for coactivator peptides, even in absence of E2. This effect is less pronounced on the phosphorylated Y537 of ERα. When bound to E2, the phosphorylated ERα actually reduces the affinity for some coactivator peptides. These differences in subtype behavior were associated with greater stabilization of the H12 ERβ due to a salt bridge formed between K300 and the phosphorylated Y488. This salt bridge is indeed likely to be stronger than the one between K300 and D303 (intrahelical) that is present the wt ERβ because of the extra negative charge of the phosphate group.

### Alternative H12 position corresponds to a stable conformation in free-energy landscape and could explain the low activity of ERβ LBD

ERβ has lower transcriptional activity than ERα at equal concentrations of E2. This difference could be attributed to the AF-1 and AF-2 regions of the receptors, located in NTC domain and LBD, respectively. The reasons why ERβ is less active than its counterpart are not clear, since both ER subtypes have similar binding affinities for E2^19^. In addition, the residues in the pocket of the AF-2 region are 100% conserved^18^ despite differences in the subtype affinity for coactivadors^34, 35^. The results presented in the previous section show that the stability of the H12 active canonical position seems to be an important difference between the subtypes. Furthermore, our alternative structure of ERβ LBD-E2 indicates that even bound to an agonist ligand such as E2, this receptor can adopt antagonist-like conformations. If this new alternative structure would be more stable than the canonical one, it could explain why ERβ LBD has lower levels of activity than the α subtype.

A comparative analysis of the alternative and canonical ERβ LBD-E2 conformations alone does not seem to be sufficient to conclude which one of them is more stable. Both structures have equivalent hydrophobic interactions as well as one important hydrophilic interaction, namely: D303-H498 in the alternative conformation (Figs 1 and 2B) and K480-N496 in the canonical structure (Fig. 4A). Participation of H498 in such a key interaction introduces additional complicating factors since this residue can adopt three different protonation states, depending on its local chemical environment, its pKa value and the intracellular pH. The estimated pKa of H498 is 6.9 in the alternative conformation and 4.8 in canonical one, indicating that under physiological pH (~7.3–7.4) the uncharged histidine is the main protonated state in both conformations. The local interaction with D303 in the alternative conformation shows H498 with a hydrogen atom in the epsilon nitrogen (hereby referred to as H498e). Small variations of the intracellular pH can change the favored protonation state, especially in the alternative conformation, with pKa near to physiological pH. Thus, the fully charged state of H498 (H498+) cannot be ignored.

**Figure 5:**
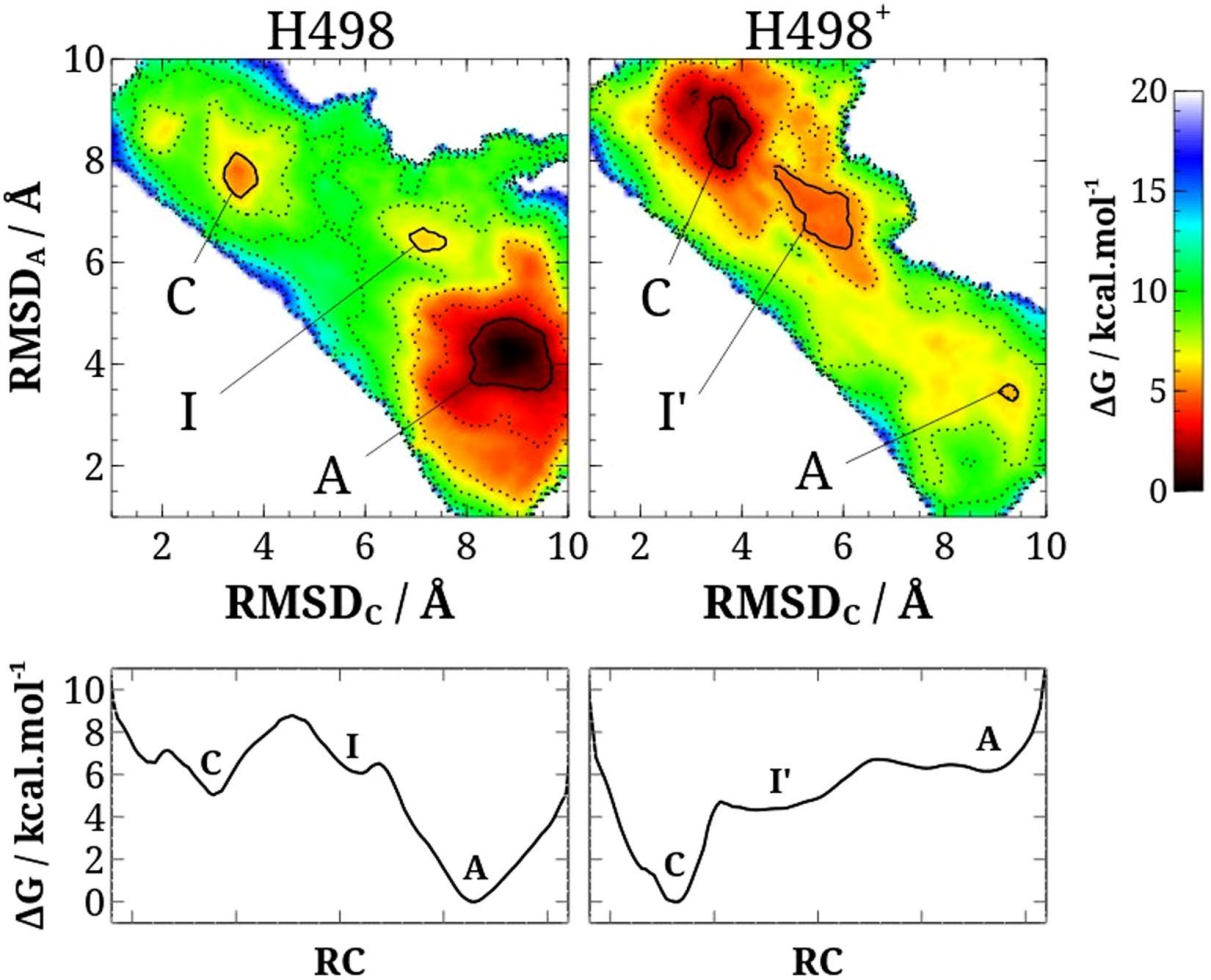
Free-energy landscape of H12 conformation: (**A**) Free-energy maps of H498e and H498+ protonation states of ERβ LBD-E2 structure. The free energy values are shown in a color scale and dashed contour lines (ranges of 2 kcal. mol^−1^). The regions of global and local minimum free-energy are highlighted with solid lines and identified as: alternative-like conformations (**A**), canonical-like conformations (**C**) and intermediates (I). (**B**) Minimum free-energy paths along a generalized reaction coordinate (RC) of H498e and H498+ protonation states of ERβ LBD-E2 structures, obtained from the free-energy maps.

To answer which H12 conformation is more stable, we carried extensive MD simulations using the adaptive biasing force (ABF) method^36–38^ to compute the conformation free-energy landscape of the ERβ LBD-E2 complex. Two H498 protonation states, H498e and H498+, were considered. In particular, we studied the free-energy landscape region involved in the transition between canonical and alternative conformations. Since there is no simple reaction coordinate that describes the transformation between these conformations, we use the RMSD relative to canonical (RMSD_C_) and alternative (RMSD_A_) crystallographic structures as reaction coordinates themselves. This multi-dimensional strategy allowed sweeping the local conformational space of ERβ LBD-E2 complex, showing which are the possible reaction pathways, intermediates, and barriers, as well as the free-energy difference between alternative and canonical conformations. The results are presented as two-dimensional free-energy landscapes in Fig. 5A. On the outset, the results clearly indicate that alternative-like conformations (A) of the ERβ LBD-E2 are favored when the histidine protonation state is H498e, whereas canonical-like conformations (C′) are stabilized for the H498+ state (i.e., under more acidic conditions).

For H498e, there is an evident global minimum located in a region around RMSD_C_ of 8.00–9.50 Å and RMSD_A_ 3.50–5.00 Å, as shown in the left panel of Fig. 5A. This region corresponds to a set of alternative-like conformation structures (labeled A in the free energy map). We also observed that the canonical-like conformations (labeled C) are not thermodynamically favored for this protonation state; the C-conformers occupy a local basin (relative depth of 5 kcal. mol^−1^) in the region around point (3.50, 8.00). The highest barrier along the minimum free-energy path connecting the global and local conformational basins is about 9 kcal. mol^−1^ and occurs near the coordinates (5.00, 8.00). The free energy landscape also suggests the existence of a weak intermediate (I) near the global basin of alternative conformations, in the vicinity of coordinates (7.25, 6.50). The structures related to this local free energy minimum are similar to ER LBD bound to antagonists (see Supplemental Figure S2). Given the characteristics of the H498e free energy landscape, we conclude that alternative conformations are predominant, being thermodynamically and kinetically favored in relation to the canonical conformation under this protonation condition.

The free energy landscape obtained when the histidine 498 is fully charged (H498+) is shown in the right panel of Fig. 5A. Interestingly, the global free energy minimum shifts to a set of canonical-like conformations (C′) located in a large area of the free energy landscape of coordinates RMSD_C_ 2.50–4.50 Å and RMSD_A_ 7.50–9.50 Å. Closer inspection of the trajectories indicates that the positively charged H498+ residue stabilizes the canonical conformation by forming a salt bridge with E332. Alternative-like conformations (A′) become a local free energy basin of 6 kcal.mol^−1^ around coordinates (9.75, 3.50). Other differences relative to the H498e protonation state occur in the transition between the conformations. H498+ exhibits a larger set of intermediate conformations (I′) near the canonical ones, around RMSD_C_ 4.50–6.00 and RMSD_A_ 6.00–8.00. Similarly to the H498e case, the intermediate I′ conformations also resemble the antagonist-like structures. They form a large basin in the free energy landscape approximately 4 kcal. mol^−1^ deep. A ΔG barrier of approximately 7 kcal.mol^−1^ separates alternative from canonical conformations when H498 is fully charged (roughly 2 kcal.mol^−1^ lower than the corresponding barrier for the H498e protonation state). For H498+, the overall basins of A′ and I′ conformers are significantly shallower than that of H498e, which allows for a more mobile and dynamic H12 in ERβ LBD-E2 in the former protonation state.

Representative views of the LBD as one moves along the path of minimum free energy between alternative and canonical structure are provided as a movie in Supplementary Information. The computed free energy landscapes are very similar to those expected in the qualitative graded agonism models^8, 15^. The distinct H498 protonation states exemplify a single-point change in the structure, similar to point mutations and phosphorylation, which could imprint stabilization of a specific conformation. Indeed, recent simulations of ERα LBD suggested that a newfound cancer-activating mutation (D538G) could reshape the energy landscape by preferential stabilization of active conformations^39^. Since H498e is the most likely protonation state, according to the pKa estimates, we conclude that our alternative crystallographic structure of ERβ LBD-E2 represents a stable conformation of the receptor. This result provides an appealing rationalization of the reduced activity of ERβ LBD in the presence of E2. Coactivators binding could further alter H12 conformation equilibrium since their association might change the ERβ LBD free energy landscape. However, the affinity for the coactivator has to compensate the free energy gain between the distinct, locally stable ERβ LBD conformations. Hydrophilic interactions between H12 and the remaining LBD in ERα (Fig. 4A) may compensate eventual effects of the protonation state of H547 (corresponding to H498 in the ERβ subtype), with the canonical structure being the thermodynamically favored conformation for this ER subtype. Recent MD simulations suggest that dimerization can also favor the ERα H12 agonist position^10^.

H498 seems to be a key factor underlying the mechanism of allosteric interactions of the ERβ LBD and their implications to its activity. The foreseeable functional role of this histidine residue is also fascinating because its protonation state could be modulated by intracellular pH. Tissues with distinct intracellular pHs will have different proportions of H498+ and H498e states and, therefore, the fraction of ERβ receptor in active canonical-like conformations could be tuned by the pH of the local environment. Our results indicate that the activity of ERβ LBD-E2 should depend on the local pH. In tissues with low intracellular pH (more acidic), such as in cells of bone tissues^40^, a greater activation of ERβ would be expected. In neutral to basic intracellular pH, such as in tumor cells^41, 42^, our results predict lower activation of ERβ by E2 due to the competing antagonism of the alternative conformation of H12. This is exactly the behavior of some selective estrogen receptor modulators (SERMs), ligands which promote agonist or antagonist actions depending on the tissue^43, 44^. These results open new opportunities for further site-directed mutagenesis and transactivation assays studies aimed at investigating ERβ activity modulation under different physiological pH conditions.

ERβ LBD alternative structure provides new insights into how ER antagonists can be rationally designed. According to the classical view of ER modulators, antagonists can occupy the same ligand cavity filled by agonists. However, antagonists are usually large molecules that sterically disrupt H12 active/canonical conformational. For instance, piperidyl group of raloxifene promotes repositioning of H12 to the coactivator-binding groove^13, 18^. On the other hand, ER agonists frequently do not even promote any contacts with H12 (for example the endogenous E2 displayed in available crystal structures^25^). One might argue that current ERβ LBD structure with H12 in the alternative conformation indicate a new possibility of designing novel β-selective antagonists via productive interactions with H12, favoring the alternative conformation. Slight adaptations in L490 (H12 helix) and M487 (loop between H11-H12) residues induced by interactions with small side-chain extensions of the ligands could make possible direct H-bond interactions with E493 (H12). These interactions might stabilize the alternative conformation and block one of the residues of the charge clamp (E493) at the same time. Since the active/canonical H12 conformation in ERα LBD seems to be more stable, its cavity should be less adaptable, reducing the affinity of this hypothetical antagonist ligand to ERα subtype of the receptor as compared to ERβ LBD.

The differences between ERα and ERβ in terms of their activity are very intriguing. For example, it is known that ERα and ERβ can result in contrasting transcriptional effects when binding to the AP-1 response element, with ERα stimulating the transcriptional activity when bound to estradiol and ERβ repressing the transcriptional activity when bound to the same ligand^45^. One proposed mechanism for this marked difference in the AP-1 site, the AF-1 independent mechanism, involves the recruitment of corepressors by the ligand bound receptor. Under such scenario, the complex ER-NCoR could recruit repressor components such as HDAC, taking these components out of the promoters regulated by the AP-1 site^46^. It is tempting to speculate that for an estradiol bound ERβ, this mechanism must involve a structural rearrangement of the H12, which would prevent the recognition of coactivators and allow the recruitment of corepressors. The alternative antagonist-like conformation of the ERβ bound to estradiol shown here might play an important role in this mechanism.

In summary, here we determined a new ERβ LBD-E2 structure in which H12 adopts an alternative antagonist-like conformation. Except for H12, which is oriented in the opposite direction as compared to the canonical ER structures in active conformation, the present structure is very similar to the previously published structures of ERs LBD. Extensive MD simulations indicate that, like the canonical H12 orientation, the alternative ERβ H12 position also corresponds to a stable conformation in solution and the protonation state of H498 dictates which of the two conformational states is predominantly stabilized depending on the pH conditions of the environment. Our results provide new perspectives on the mechanisms of ERs action, shedding light on the putative molecular basis for the lower activity of ERβ LBD.

## Materials and Methods

### Protein purification and crystallization

The human estrogen receptor LBD (residues 256–501) cloned in the pSMT3 vector was expressed in the *E. coli* strain BL21 (DE3)RIL at 20 °C overnight. Cell pellets of over-expressed hERβ were lysed in a buffer 50 mM Tris-HCl pH 8.0, 300 mM NaCl, 5 mM imidazole, 10% glycerol and 10 mM beta-mercaptoethanol supplemented with 1 μM 17 β-estradiol. The complex hERβ-estradiol was purified on a NiNTA affinity column (Qiagen) and dialyzed overnight into 20 mM Tris–HCl (pH 7.3), 150 mM NaCl, and 10 mM β-mercaptoethanol in the presence of the SUMO protease Ulp1 (Ubl-specific protease 1). The protein was further purified by size exclusion chromatography on a Superdex-75 (16/60) column (GE), equilibrated with the dialysis buffer. The pooled peak fractions corresponding to the complex hERβ-estradiol were analyzed by SDS-PAGE and native PAGE gels and concentrated to 5 mg/mL, using a centrifugal concentrator with a polyethersulfone membrane with a 10,000 MWCO (VIVASPIN, Sartorius Stedim Biotech). Crystallization drops of 1 μL volume, using a 1:1 protein/mother liquor ratio, were set up automatically (Honeybee 931, Genomic Solutions Inc) in 96-well sitting-drop plates and allowed to equilibrate at 19 °C. One diffracting-quality unique crystal was obtained in the presence of 0.1 M Na-citrate (pH 5.5), 10% [w/v] PEG-4000, 10% [v/v] isopropanol appeared within 2 weeks and it was used for X-ray data collection. The crystal, containing a dimer of hERβ LBD per asymmetric unit, belongs to the trigonal space group P3_2_22 with unit cells dimensions a = b = 83.8 Å and c = 168.5 Å.

### X-ray structure determination

The X-ray diffraction dataset was collected from a single crystal at 2.5 Å resolution on MX2 beamline of the Brazilian National Synchrotron Light Laboratory^47^. The diffraction data were processed using the program XDS^48^. The crystallographic structure was determined by molecular replacement with the program Phaser^49^ using the phosphorylated LBD structure of estradiol bound hERβ (PDB code 3OLL) as a template. Non-covalently bound water molecules and ligand were removed prior molecular replacement. The 2F_o_ − F_c_ map calculated from the unique solution of the top rotation and translation searches showed a clear and contiguous electron density for the protein and the ligand estradiol originally not included in the search model. Model building and refinement was carried out using the program Coot^50^. Cycles of restrained refinement with the program Refmac5^51^ were initially carried out using overall temperature factors and later on isotropic temperature factors. One cycle of simulated-annealing was applied with the program Phenix^52^. Water molecules were added using the program Arp/wArp^53^ and the stereochemical quality of the protein complex was validated with the program MolProbity^54^. Statistics of the refined structures are presented in Table S1 of the Supplementary Information. The complete ERβ LBD polypeptide chains, except for the first five and six N-terminal residues of chains A and B respectively and residues at the loop region connecting H9 and H10 (residues 412 to 419 from chain A and 413 to 420 from chain B), are clearly visible in the final homo-dimeric ERβ structure. The refined model of the asymmetric unit comprises the physiological dimeric assembly of the ERβ (Supplemental Figure S3), where the dimerization interface is formed mainly via hydrophobic interactions and H-bonds between residues of H10 and H11^13^. The protein and ligand interactions observed in chains A and B of the dimer were basically the same. Consequently, the two complexes ERβ LBD-E2 present in the crystal asymmetric unit will be treated hereafter as a unique model.

### Molecular dynamics simulations

Five human ER LBD systems were prepared for the MD simulations, namely: i) alternative crystallographic structure of wild-type ERβ LBD with H498 in the uncharged protonation state (hydrogen in epsilon nitrogen, H498e); ii) alternative crystallographic structure of wt ERβ LBD with H498 in the charged protonation state (H498+); iii) canonical crystallographic structure of wild-type ERβ LBD (PDB code 3OLS); iv) canonical structure of mutant ERβ LBD K300N (K300Nβ), prepared *in silico*; v) canonical structure of wt ERα LBD (PDB code 1QKU). The complete simulated systems were built with Packmol^55^ and contained the LBD, E2, structural water molecules and a 15 Å thick solvation layer with around the protein consisting of at least 16000 water molecules and counter ions for electroneutrallity. The missing coordinates were modeled by I-TASSER^56^. Histidines pKa and their protonation states were estimated with PROPKA^57^. After minimization and equilibration procedures, each entire system was simulated for 100 ns in triplicates independently. All MD simulations were performed with NAMD^58^ at 298 K and 1 bar, using CHARMM parameters for proteins^59^ and the TIP3P model for water^60^. E2 was parameterized in a previous study^61^. Protein stability during the MD simulations was verified via calculation of backbone RMSD as compared to the crystallographic structures (Supplemental Figure S4).

### Free-energy landscape calculations

The free-energy difference between the ERβ LBD conformations was investigated using as reaction coordinates the RMSDs in relation to the α-carbon atom positions of the residues 469 to 501 (carboxy-terminus of H11, H11-H12 loop and H12) in the canonical (RMSD_C_) and alternative (RMSD_A_) crystallographic structures. Part of this peptide corresponds to the region that changes more its position between the studied ERβ LBD conformations. The use of reaction coordinates based on the RMSD of the region of the protein known to be important for the conformational change provides a more accurate description of the process pathways^11, 62^. Each RMSD coordinate covered a range of 1 to 10 Å. The free energy differences - ΔG (RMSD_C_, RMSD_A_) - was determined using MD simulations coupled with the Adaptive Biasing Force (ABF) method, implemented in NAMD^58^. Instantaneous values of the force were accrued in bins 0.01 Å^2^ wide (0.1 × 0.1 Å). Boundary potentials with a force constant of 10 kcal.mol^−1^. Å^−2^ were also used. The minimum number of steps sampled (N_samples_) before the full application of ABF force was equal to 4000. These parameters appear to constitute reasonable choices in other free energy calculations using ABF method^36–38^. MD simulations conditions and parameters were the same as described in the previous section. Two systems are studied: i) ERβ LBD with H498e protonation state; ii) ERβ LBD with H498+ protonation state. For each system, 14 independent ABF MD simulations were carried out for 100 ns, totaling 1.4 μs each system. The initial structures were obtained from the equilibrium MD simulations described above. One half of the ABF simulations initiated from the canonical ERβ LBD conformation and the other half initiated from the alternative structure. The free energy landscape map of each system was calculated as the average over all simulations by weighting the samples by dG/dr from each simulation bin. As H12 conformations were sampled during ABF MD simulations, backbone RMSD not including this helix was used as a measure of the overall LBD stability. The analyses show RMSD values around 0.9–3.5 Å (independent of reference structure being canonical or alternative ERβ LBD), confirming that ERβ LBD structures maintain themselves stable along the trajectories (Supplemental Figure S5).

### Data availability

The coordinates and structure factors have been deposited in the Protein Data Bank under the accession code 5TOA.

## Electronic supplementary material

Supplementary Information file

Representative views of ERβ LBD movie
