# Supplementary material for: An alternative conformation of ERβ bound to estradiol reveals H12 in a stable antagonist position
Source: Sci Rep. 2017 Jun 14;7:3509. doi: 10.1038/s41598-017-03774-x (PMC5471280; doi:10.1038/s41598-017-03774-x)
Supplement: Supplementary file 1 — Supplementary Information file [file 41598_2017_3774_MOESM1_ESM.pdf]

Supplementary Information for:

An alternative conformation of ERβ bound to estradiol reveals H12 in a stable antagonist position

Paulo C. T. Souza[1,2]*, Larissa C. Textor[3]*, Denise C. Melo[1], Alessandro S. Nascimento[3], Munir S. Skaf[1] and Igor Polikarpov[3]

[1]Institute of Chemistry, University of Campinas - UNICAMP, P. O. Box 6154, Campinas, SP, Brazil; [2]Groningen Biomolecular Sciences and Biotechnology Institute and Zernike Institute for Advanced Materials, University of Groningen, Nijenborgh 7, 9747 AG Groningen, The Netherlands; [3]Physics Institute of São Carlos, University of São Paulo - USP, P. O. Box 396, São Carlos, SP, Brazil.

*These authors contributed equally to this work.

Content:

1. Supplementary Table S1 X-ray refinement statistics
2. Supplementary Figures
   a. Figure S1. Alternative H12 is partially overlapped with coactivator groove.
   b. Figure S2. Representative structures of the global and local minimum of free-energy landscape maps.
   c. Figure S3. Physiological homo-dimeric alternative ERβ LBD-E2 structure.
   d. Figure S4. Overall stability of ER-LBD structures during conventional MD simulations.
   e. Figure S5. Overall stability of ER-LBD structures during ABF MD simulations.

Tables

Table S1. X-ray data collection and refinement statistics

**Resolution range**	41.91 - 2.5 (2.59 - 2.5)
**Space group**	P 3_2 2 1
**Unit cell**	83.811 83.811 168.531 90 90 120
**Total reflections**	157,355 (15,493)
**Unique reflections**	24,355 (2,389)
**Multiplicity**	6.5 (6.5)
**Completeness (%)**	99.76 (100.00)
**Mean I/sigma(I)**	11.88 (1.62)
**Wilson B-factor**	56.41
**R-merge**	0.1027 (1.285)
**R-meas**	0.1117 (1.394)
**R-pim**	0.04344 (0.5345)
**CC1/2**	0.997 (0.575)
**CC***	0.999 (0.854)
**Reflections used in refinement**	24,352 (2,391)
**Reflections used for R-free calcualtion**	1,198 (106)
**R-work**	0.2052 (0.3158)
**R-free**	0.2428 (0.3492)
**CC(work)**	0.961 (0.700)
**CC(free)**	0.954 (0.599)
**Number of non-hydrogen atoms**	3664
**macromolecules**	3561
**ligands**	40
**solvent**	63

**Protein residues**	462
**RMS(bonds)**	0.002
**RMS(angles)**	0.61
**Ramachandran favored (%)**	97.12
**Ramachandran allowed (%)**	2.88
**Ramachandran outliers (%)**	0.00
**Rotamer outliers (%)**	0.51
**Clashscore**	4.96
**Average B-factor**	64.50
**macromolecules**	64.80
**ligands**	50.46
**solvent**	56.32

[a] Values in parentheses are for the highest resolution shell.

[b] $R_{merge} = \Sigma \mid I_j - \langle I \rangle \mid / \Sigma \langle I_j \rangle$, where I_j is the observed intensity of an individual reflection and $\langle I \rangle$ is the average intensity of that reflection.

[c] Root-mean-square-deviation.

Figures

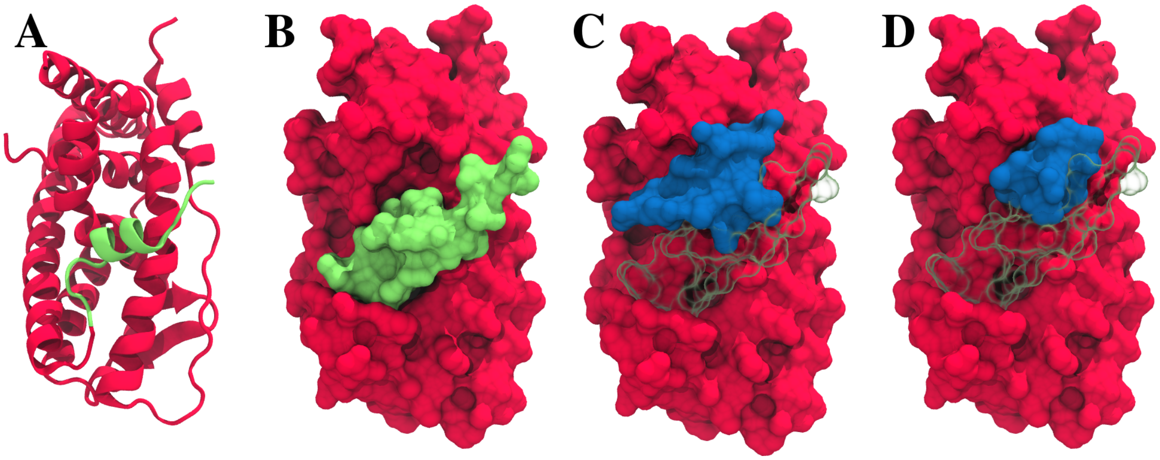

Figure S1. Alternative H12 conformation partially overlaps with the coactivator groove. Alternative ERβ LBD-E2 conformation shown as cartoon (**A**) and in surface representation (**B**) with H12 highlighted in green and the rest of LBD structure in red. Alternative ERβ LBD-E2 structure superimposed with the canonical ERβ LBD in complex with E2 (PDB id: 3OLS) and KB095284 (PDB id: 4ZI1) ligands bound, respectively, to coactivator SRC1 Box2 (**C**) and CIA12 coactivator peptide (**D**) displayed as blue surfaces.

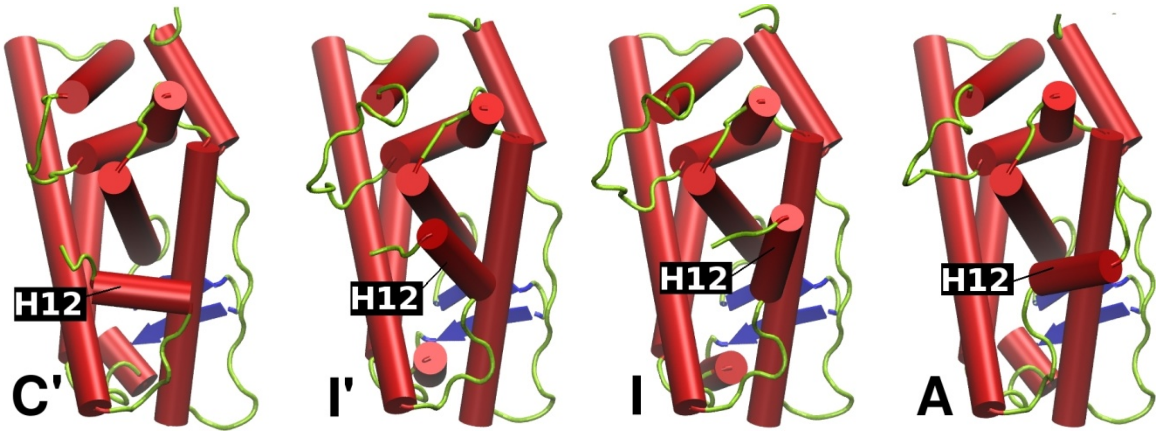

Figure S2. Representative structures of the global and local minimum of free-energy landscape maps.

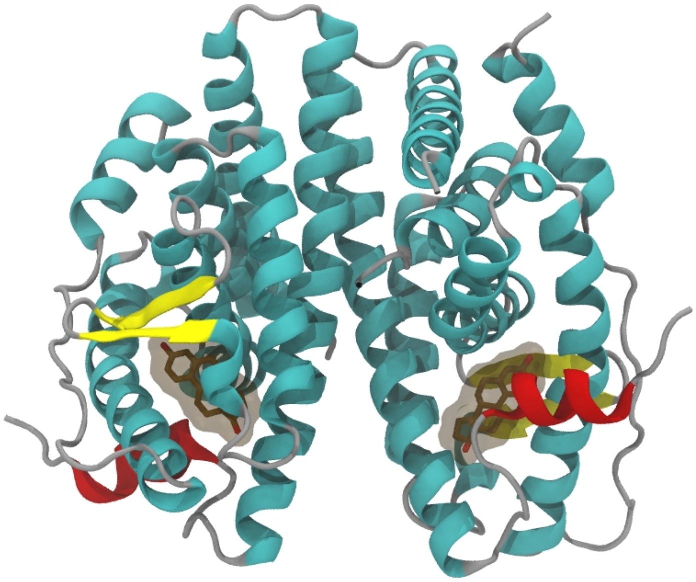

Figure S3. Physiologically relevant homo-dimeric ERβ LBD-E2 structure in alternative conformation.

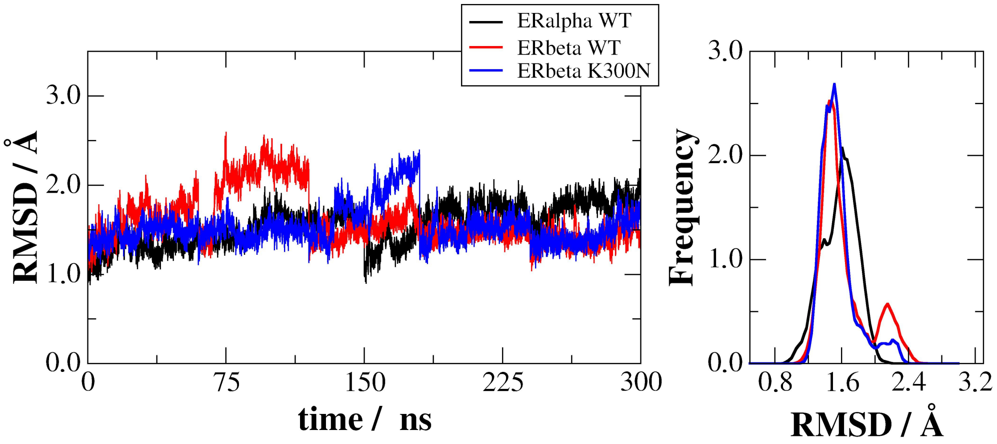

Figure S4. The overall stabilities of ER-LBD structures during the conventional MD simulations were verified via the calculations of backbone RMSD with respect to the canonical crystal structures (PDB id: 1QKU for wild-type ERα LBD-E2 and PDB id: 3OLS for wild-type and K300N mutant of ERβ LBD-E2). The results are displayed in

plots of RMSD as a function of time (with three 100-ns concatenate trajectories for each system) and RMSD distributions.

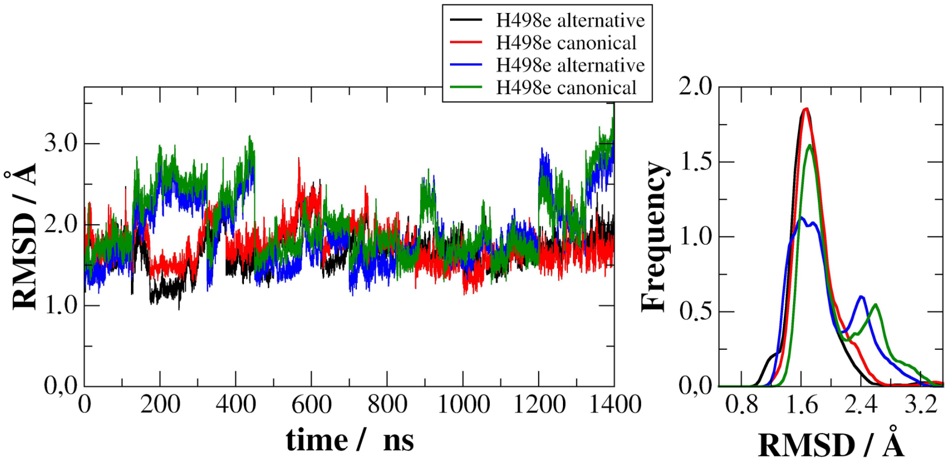

Figure S5. The overall stabilities of LBD structures during ABF MD simulations (in H498e and H498+ protonation states) were verified via the calculations of backbone RMSD with respect to the canonical (PDB id: 3OLS) and the alternative ERβ LBD-E2. Since H12 conformations were biased ensemble sampled during these simulations, they were not included in this analysis. The results are displayed in plots of RMSD as function of time and RMSD distributions.
